# Laboratory assessment of sensitive molecular tools for detection of low levels of *Echinococcus multilocularis*-eggs in fox (V*ulpes vulpes*) faeces

**DOI:** 10.1186/1756-3305-7-246

**Published:** 2014-05-28

**Authors:** Øivind Øines, Mats Isaksson, Åsa Hagström, Saraya Tavornpanich, Rebecca K Davidson

**Affiliations:** 1 Norwegian Veterinary Institute, Post boks 750 Sentrum, 0106, Oslo, Norway; 2 National Veterinary Institute, Uppsala 75189, Sweden

**Keywords:** *Echinococcus multilocularis*, Surveillance tool, Real-time PCR, Molecular detection, Replicates

## Abstract

**Background:**

In endemic areas with very low infection prevalence, the frequency and intensity of *Echinococcus multilocularis* can be extremely low. This necessitates efficient, specific and sensitive molecular tools. We wanted to compare the existing molecular tools, used in the Norwegian national surveillance programme, and compare these with new techniques for detection of this zoonotic pathogen in fox faeces. Here we present the results of screening samples containing a known level of *E. multilocularis* eggs with two highly sensitive DNA isolation and extraction methods combined with one conventional PCR and three real-time PCR methods for detection.

**Methods:**

We performed a comparison of two extraction protocols; one based on sieving of faecal material and one using targeted DNA sampling. Four methods of molecular detection were tested on *E. multilocularis*-egg spiked fox faeces.

**Results:**

There were significant differences between the multiplex PCR/egg sieving DNA extraction methods compared to the new DNA fishing method and the three real-time PCR assays. Results also indicate that replicates of the PCR-reactions improve detection sensitivity when egg numbers are low.

**Conclusions:**

The results indicate that the use of real-time PCR combined with targeted DNA extraction, improves the sensitivity of *E. multilocularis* detection in faecal samples containing low numbers of *E. multilocularis* eggs. Results also indicate the importance of replicates of the PCR-reactions when pathogen levels are low.

## Background

Effective, sensitive surveillance tools are important to help determine the distribution of pathogens. *Echinococcus multilocularis* eggs are zoonotic and can cause fatal hydatidosis in humans [1]. Annual surveillance programmes in mainland Norway and Finland, in suitable host species (red fox (*Vulpes vulpes*) and raccoon dog (*Nyctereutes procyonis*)), suggest that *E. multilocularis* is currently absent, or at least below the estimated required detection threshold of 1% [2,3]; **EFSA zoonoses report** [http://www.efsa.europa.eu/en/scdocs/scdoc/223r.htm]. This zoonotic cestode continues to push at its northernmost distribution boundaries [4-6]. *Echinococcus multilocularis* was first detected in mainland Fennoscandia in Denmark in 2005 [7] and in neighbouring Sweden in 2010 [8] and has been detected on the Norwegian high-Arctic island of Svalbard [9]. In Sweden, only four of the 2985 foxes examined during 2011 surveillance programme harboured *E. multilocularis* worms [10], highlighting the extremely low prevalence of this parasite in this region (0.1%). Recently, *E. multilocularis* has been detected in four foxes from Denmark in a study of 679 wild carnivores, indicating a countrywide *E. multilocularis* prevalence of only 0.7% [11]. It is vital that the methods used in surveillance programs are able to detect this very low prevalence, especially in areas where the burden of infection is expected to be low. The methods used in surveillance programs must not only be highly sensitive and specific, but also cost-effective so as to allow for high sample throughput to enable sufficient numbers to be screened.

The current gold standard method for the detection of *E. multilocularis* in the definitive-host is intestinal examination (sedimentation counting technique (SCT)) using microscopy. This method is purported to have close to 100% sensitivity and specificity [12]. We argue that this approach is very laborious and costly due to the high labour costs involved in the analysis. Additionally, SCT must be performed by specially trained staff. This approach is not feasible in countries where infection levels are assumed to be very low or absent and, labour costs are high. Another approach for *E. multilocularis* detection is through coproantigen detection [12] as is used in the surveillance program in Belgium [13]. Coproantigen tests have the potential to detect antigens prior to patency, before eggs are excreted in the faeces. False negative and false positive results obtained in coproantigen tests are problematic for surveillance programmes in regions considered to be free from *E. multilocularis*. Coproantigen detection has been shown to have high sensitivity when worm burdens are moderate to high, but the occurrence of false negatives when worm burdens are lower (50 or less) [14] is of concern. All four of the Danish *E. multilocularis* positive foxes harboured less than 27 adult worms [11], highlighting the need for sufficiently sensitive diagnostic tools in surveillance programmes. Surveillance programs in Norway and Great Britain have chosen to use molecular methods for the detection of *E. multilocularis* DNA in faecal samples [2,15]. The majority of approaches rely on the physical isolation of eggs followed by DNA isolation to decrease the presence of background DNA or inhibitors in faecal samples. In Norway, fox faecal samples have been analysed since 2006 using egg-isolation, followed by DNA-extraction and detection using a multiplex PCR for the identification of the *E. multilocularis* eggs [2]. The method used for the screening of most of these samples was initially described by Trachsel and colleagues [16]. The results of the 2780 fox faecal samples screened to date indicate that the tapeworm is either absent, or present in very low numbers in Norway, given that no positive fox samples have yet been detected [17]. A disadvantage of the mPCR method are the multiple steps performed prior to DNA extraction: including the physical sieving of eggs from the faecal material and alkaline lysis and proteinase K digestion of the material prior to DNA-extraction using conventional spin columns. Not only is this ‘sieving’ approach time consuming and labour intensive, when compared to traditional DNA-extraction protocols, but the sieving stage is also vulnerable to blockage from faecal particles, potentially preventing eggs passing through the first sieving stage. Broken eggs might also be flushed through the second nylon mesh and this DNA-material can be lost. Additionally, this approach only targets eggs and any remaining parasite material in the faeces may therefore remain undetected. It would be preferable to have parasite DNA-extraction systems where high volumes of fox faeces can be analysed and a variety of parasite material, such as eggs and proglottids, could easily be detected.

Real-time PCR methods have become available for *E. multilocularis* detection [18]. Real-time PCR further reduces the time and cost of diagnosis, as well as increasing sensitivity [19-21]. Real-time PCR is performed in a closed tube system thus minimizing the risk for cross-contamination and operational error given fewer manual steps. Real-time PCR also has the potential to indicate the relative amount of target DNA present in a sample, based on comparisons between cycle thresholds (C_t_) from several samples. Development of real-time PCR assays for *E. multilocularis* detection, which can identify low amounts of DNA in faeces from foxes, may improve detection sensitivity. The sensitivity of the methods used in a surveillance program will not only influence the quality of the results but also affect the number of samples required to achieve a minimum detection level.

**Figure 1:**
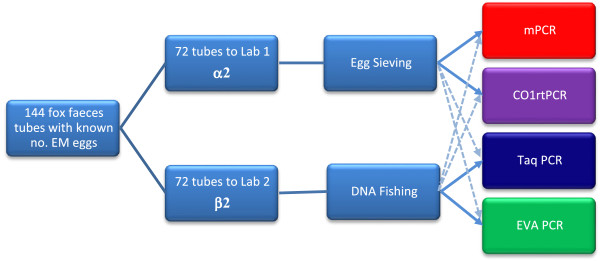
**A flow diagram outlining the experimental design of samples containing fox faeces with a known number of *Echinococcus multilocularis *eggs (α2 and β2).** 144 samples were divided into two batches for DNA extraction at two different laboratories. Each of the laboratories then carried out two of the four PCR methods on the DNA extractions done at their laboratory (solid line) as well as on DNA extractions sent from the other laboratory (dotted line).

In this study we wanted to compare different DNA isolation methods and molecular detection methods which were available in our laboratories and try to identify the best combination for use in future surveillance programs (Figure 1). The primary aim of this study was to compare the performance of two sample preparation protocols; the egg-isolation and DNA extraction used in Davidson *et al.*[2] ‘sieving’, with a new DNA isolation procedure ‘DNA-fishing’ using fox faeces spiked with eggs from *E. multilocularis*. A secondary aim was to compare a selection of real-time PCR methods for *E. multilocularis* detection. Comparison of the PCR systems were performed in a blinded trial using two batches of DNA samples prepared from spiked fox faecal samples containing a known number of *E. multilocularis* eggs. The use of spiked material was a cost-effective alternative to the use of real-life endemic samples identified by necropsy and sedimentation and counting technique. Laboratory spiking of pooled fox faeces allows almost identical samples to be analysed in parallel. The ability to select samples with very low levels of eggs may also help address the sensitivity and reproducibility of different methods more precisely. In comparison the exact egg numbers would be unknown in samples from naturally infected foxes. Results from these experiments helped with the selection of optimised methods for a surveillance programme in an *E. multilocularis* ‘free’ country, such as Norway.

## Methods

### Preparation of *Echinococcus multilocularis* eggs

All preparation of *E. multilocularis* eggs and spiked samples was carried out at Laboratory 1 using inactivated eggs generously donated by Dr Thomas Romig (University of Hohenheim) which had been frozen for more than 3 days at below -80°C, and kept in ethanol. Eggs were transferred from the storage container to a plastic Petri dish containing physiological saline. Pieces of plastic (max. 2 mm × 2 mm) were placed on a glass slide and small droplets of physiological saline containing eggs was pipetted onto the surface of the plastic using a thin glass Pasteur pipette with a modified melted tip. This allowed the precise pipetting of low numbers of eggs and minimized the volume of storage solution to be transferred with the eggs, keeping any free DNA in solution or attached to the egg surface to minimal levels. After being placed on the plastic film, egg counting was carried out at 40 × magnification using a stereomicroscope. Each small piece of plastic had a known number of eggs and these plastic pieces were carefully individually transferred into labelled sample tubes: Eppendorf tubes or 15 ml Falcon tubes, depending on the extraction protocol which followed.

### Preparation of ‘*E. multilocularis* -egg only’ series

Two batches of eggs were prepared without faeces (α1/β1). Each batch was made up of 24 samples, in either 1.5 ml Eppendorf tubes (α1) or 15 ml falcon tubes (β1), depending on protocol and each tube contained a small piece of plastic with a known quantity of eggs suspended in physiological saline. Samples in batch α1 and β1 contained 1-24 eggs (Additional file 1: Table S1).

Batch ‘α1’ was DNA extracted using ‘sieving’, according to Davidson *et al.*[2] at Laboratory 1, starting from the alkaline lysis step and protein K digestion, followed by the protocol previously reported. Batch ‘β1’ was extracted using a simplified^a^ targeted DNA extraction method known as ‘DNA-fishing’, an *in-house* protocol developed at laboratory ‘2’. This method uses a capture probe system: streptavidin coated magnetic beads which attach to a biotinylated DNA capture probe complementary to *Echinococcus* mtDNA. This complex is then physically drawn out of the homogenised solution using powerful magnets. This allows, more or less, specific target DNA to be concentrated and removed from a solution containing inhibitory substances and “background” DNA, which could inhibit amplification. This targeted approach to DNA-isolation has previously been reported for detection of *Toxoplasma* from meat samples [22] and *Mycobacterium* in clinical specimens [23].

### Preparation of the spiked fox faeces batches

**Figure 2:**
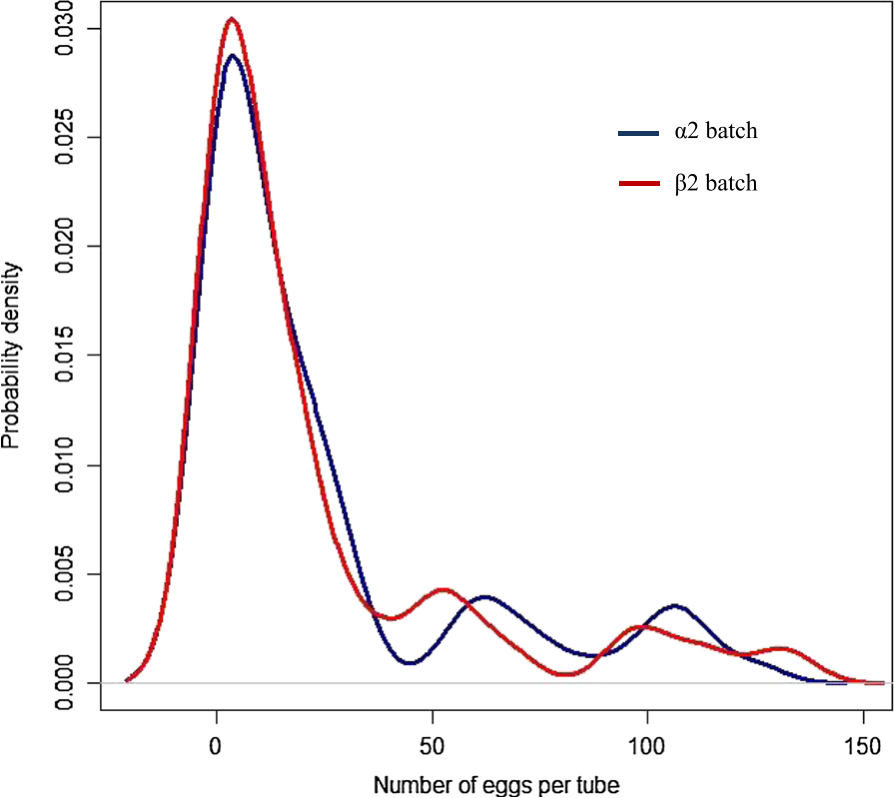
**Comparing the density distribution of egg numbers from samples from batches *α2- *egg sieving and *β2 *DNA-fishing.** Kolmogorov-Smirnov (KS) goodness of fit test was used to determine if egg distributions between the two batches, α2 and β2, were similar.

A pool of fox faeces from 29 animals, which had been confirmed as *E. multilocularis* negative during the Norwegian 2010/2011 surveillance programme, was homogenised using vigorous stirring for five minutes. Larger debris such as hair, bones, feathers and plastic were removed from the faecal pool. One hundred and forty four 15 ml falcon tubes were filled with 3 ± 0.05 g of the homogenised fox faeces and labelled 1 to 144 (Additional file 1: Table S2). The tubes were divided into two batches (α2/β2) (Figure 1). A known number of *E. multilocularis* eggs were added to randomly selected numbered-tubes. Fifteen tubes in each batch had 1-4 eggs added, fifteen had 5-15 eggs added; another 15 tubes had 16-50 eggs added; 51-150 eggs were added to 14 tubes; twelve tubes had no eggs added, whilst a single tube in each batch respectively contained a high number of eggs (600-1000). Density plots for the distribution of the number of eggs per tube for the α2 and β2 batches are presented in Figure 2. The Kolmogorov-Smirnov (KS) goodness of fit test was used to determine if the egg distributions between the two batches were similar [24].

### Batches α2/β2 *E. multilocularis* -DNA extractions from spiked faecal samples

Seventy-two samples of batch α2 (SID1-72) were prepared in laboratory 1, according to Davidson *et al.*[2]. Extraction of batch β2 (SID73-144) samples was performed at laboratory 2, according to Isaksson and colleagues’ DNA capture method (Isaksson, pers. comm.). As described above, the extraction protocol is a sequence-specific magnetic capture method, where DNA-probes conjugated with biotin and streptavidin are coupled with paramagnetic beads and are extracted out of the sample. The method is similar to that which has been described for *Toxoplasma* detection in meat [22]. Samples were stored either at 4°C or frozen (<-18°C) for extended periods between each analysis. The last analysis was performed no more than 7 months after DNA template preparation. Additional file 1: Table S2 provides details on the number of eggs present in each sample.

### Molecular detection of *E. multilocularis* -DNA

In this study four molecular assays were used to detect the presence of *E. multilocularis* in the DNA extraction: a conventional multiplex PCR assay and three different real-time assays (Figure 1). All the molecular methods used targeted mtDNA genes, which are likely to be present in higher numbers compared to nuclear targets. We tested one real-time PCR which used EvaGreen reporter, having no probe, and two other probe-based real-time PCR assays. These two different real-time PCR assays had different probe designs; Taqman® design with a minor groove binder (MGB)-probe and Zen™ double quenched probe; targeting *E. multilocularis* mitochondrial gene targets.

#### Multiplex PCR –mPCR

Detection of *E. multilocularis* using a multiplex PCR (‘mPCR’) was performed according to Davidson *et al.*[2]. Digital gel images were prepared using ChemiDoc™ XRS + transilluminator (Bio-Rad, Hercules CA, USA) after using GelRed™ (Biotium, Hayward CA, USA) staining and images were analysed using Image Lab software (Bio-Rad, Hercules CA, USA). Images were manually optimised in software to best visualise presence of bands indicative of *E. multilocularis*. Any bands which were present on a gel, including less intense bands were interpreted as positive. This method was performed in laboratory 1.

#### Real-time PCR MGB taqman

An MGB-taqman real-time PCR assay (‘Taq-PCR’) targeting the mitochondrial 12 s gene (Isaksson pers. comm) was run in triplicate. This method was performed in laboratory 2.

#### Real-time EvaGreen assay

An EvaGreen real-time PCR assay (‘Eva-pcr’) targeting the mitochondrial ND1 gene was designed. This assay is not specific and can also detect *Echinococcus granulosus,* necessitating confirmation using another assay. Primers ‘**Echi New F**’: 5′- CTTTCWGTRTTRTGRTTTTTAGCTG – 3′ and ‘**Echi New R**’: 5′- TACACAAAAACAAGCTTCAAACCTAAC – 3′ were used in 400 nM concentration in combination with the Bio-Rad SsoFast EvaGreen Supermix (Bio-Rad, Hercules CA, USA), giving a total volume of 15 μl, 2 μl of which were template DNA. Reactions were run in a Bio-rad CFX-96 instrument under the following conditions: 98°C for 2 seconds; forty cycles of 98°C for 2 sec, 55°C for 5 sec, plate read FAM-channel; melting curve 65°C to 85°C with 0.2°C increment, 10 sec hold and plate read under each step. Sample positive for *E. multilocularis* will generally result in a melting peak temperature of 77.2°C and *E. granulosus* 78.8°C, but melting peak temperatures may vary with samples and are therefore not conclusive. An alternative assay is necessary for confirmation. This method was performed in laboratory 2.

#### Real-time PCR using ZEN™ double quenched probe

A real-time PCR was designed (‘CO1rtPCR’) as a ZEN™ double quenched probe (Integrated DNA technologies, Coralville USA). The mitochondrial CO1-gene was selected as the target area, due to its widely accepted barcoding properties, making it, in most cases, a useful species marker. A selection of various relevant sequences available in Genbank was imported into Vector NTi (Invitrogen, Carlsbad, USA) and an alignment was constructed using the Align X module in the software package. Selected sequences from closely related species, such as *Echinococcus granulosus* were also included in the alignment, to avoid sequence regions which were conserved between species. A suitable region was found to which primers and probes were designed. Primers were ‘**EMrtCO1F**’ (5′-TGGTATAAAGGTGTTTACTTGG-3′), ‘**EMrtCO1Rew**’ (5′-ACGTAAACAACACTATAAAAGA-3′), and Zen probe: 5′-56-FAM/TCTAGTGTA/Zen/AATAAGAGTGATCCTATTTTGTGGTGGGT/3IABkFq/-3′. Real-time PCR reactions were run using Brilliant III (Agilent Technologies, Santa Clara CA, USA) mastermix. Reactions were run on the MXPro 3005x instrument, using normal two step conditions (60°C annealing temperature) and run for 40 or 45 cycles. The filter gain setting for FAM was set to eight. Results were analysed using MxPro software (Agilent Technologies, Santa Clara CA, USA). A total reaction volume of 25 μL containing 3 μL of template DNA was added into each vessel. This method was run in laboratory 1.

### Replicates to detect variation

**Figure 3:**
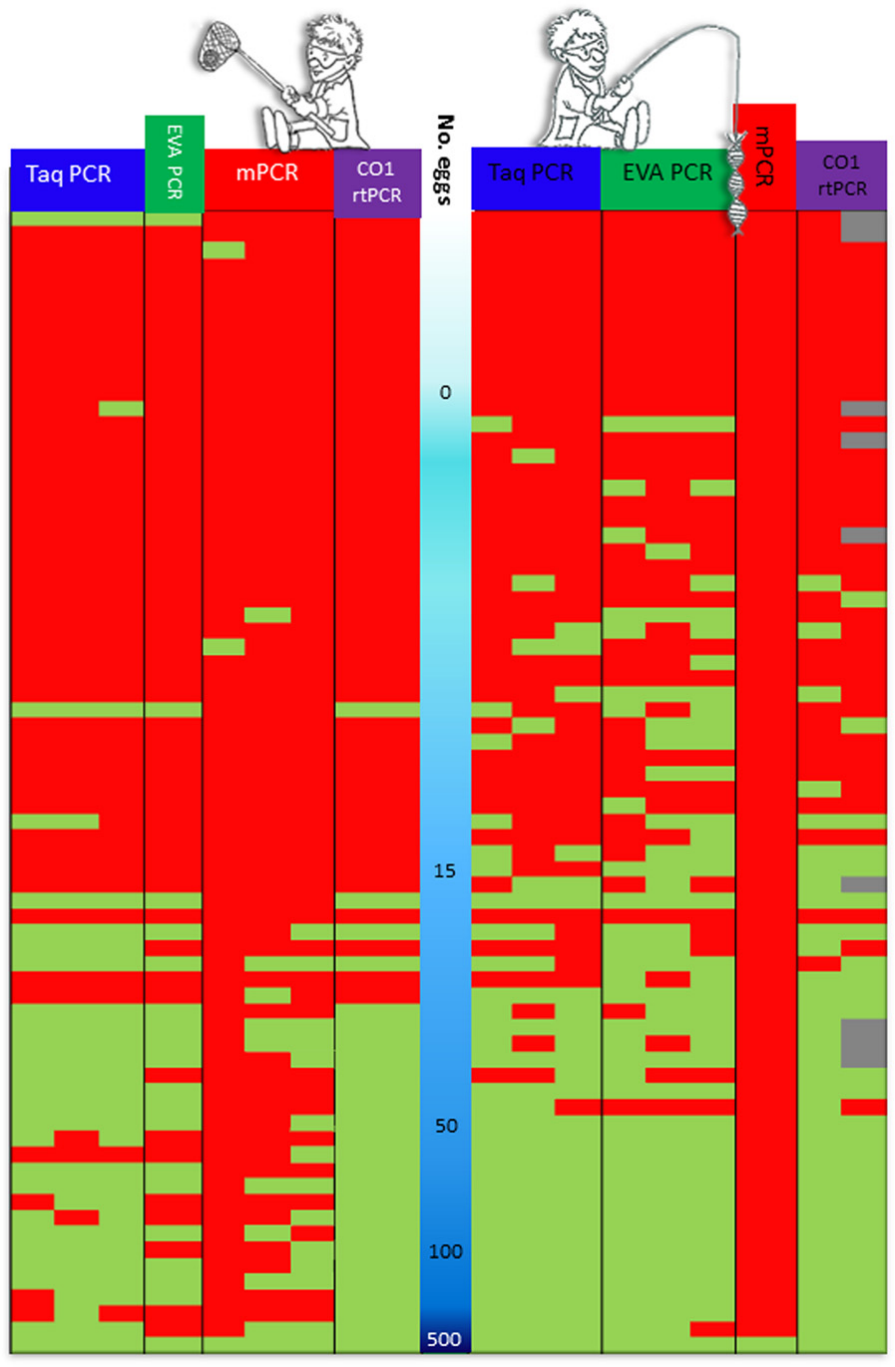
**Depicting results from batches α2 egg sieving and β2 DNA-fishing ordered by number of eggs (shown with the increasing intensity of the blue gradient from zero eggs to 500+ eggs).** Individual replicate results are marked either as negative (red) or positive (green) after being analysed using the multiplex PCR (mPCR), Taq-PCR, EVA-PCR and CO1rtPCR. Batch α2 is on the left side of the blue gradient whilst β2 is on the right side of the central blue column. Any greyed positions indicate replicates which were not available due to lack of DNA.

PCR-detection of *E. multilocularis* - DNA was planned to be carried out in duplicate for egg only batches α1 and β1, and in triplicate for batches containing spiked faeces (α2 and β2). Replicates were included to detect variation for each of the methods (mPCR/Taq-PCR/Eva-PCR/CO1rtPCR), which might be due to very low levels of target DNA. However, due to limited amounts of material available from both batches (α2 and β2) at the two laboratories during the testing, the Taq-PCR was the only method performed in triplicate for all samples. The Eva-assay was performed in triplicate for samples that had undergone the DNA-fishing extraction method but only carried out once for samples prepared by the egg sieving DNA-extraction method. The mPCR and CO1rtPCR were run in triplicate for samples prepared from ‘sieving’ (batch “α2”). Due to limited amounts of template from the ‘DNA-fishing’ (β2 batch), analysis using CO1rtPCR and mPCR was only possible in duplicate. Nine samples from the β2 batch were only analysed in a single CO1rtPCR run due to insufficient volumes of extracted DNA. The variations of replicates are represented graphically in Figure 3 and results are visible as horizontally stacked under each method.

### Statistical analysis

The different DNA extraction methods and PCR methods, in terms of test sensitivity and specificity, were compared using the receiver-operating characteristic curve (ROC) analysis and quantified by measuring the area under the curve. The approach is cut-off independent, and therefore provides a good measure of accuracy for continuous results [25].

For the present study, we plotted ROC curves and calculated area under the curves (AUC) with a 95% confidence interval (CI) for all the replicates of the four PCR methods for each of the DNA extraction methods (DNA-fishing (β2) and ‘sieving’ (α2)). The ROC presents a plot of the sensitivity versus specificity over the entire range of egg concentration. The sensitivity is defined as the probability that a method yields a positive result given that samples contain eggs. The specificity is defined as the probability that a method yields a negative result given that samples contain no eggs. The AUC, ranging from 0 to 1, presents the overall performance of the testing methods accounting for both sensitivity and specificity. We estimated AUC of each method for all samples (n = 144), and when samples only contained ≤ 15 eggs (n = 84) (Additional file 1: Table S2). The ROC and AUC s were performed using the pROC package of R software [26].

In addition, calculations of AUC using a combination of two of the three replicates from batch β2 were performed (data not shown). This was carried out to check if duplicate or triplicate analysis would influence the results.

The negative and positive predictive values were calculated in addition to the sensitivity and specificity [27]. The datasets were analysed using JMP 9.0.0 statistical software (SAS Institute Inc, NC USA) and a significance level of p < 0.05 was selected for analysis purposes.

## Results

### α1/β1 -DNA extraction assessment of *Echinococcus multilocularis* eggs only

**Figure 4:**
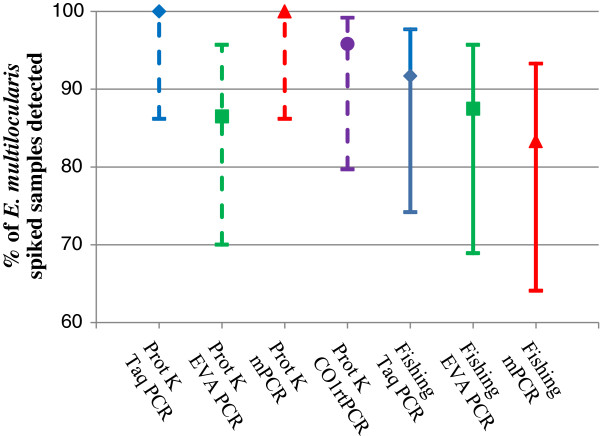
**Results from analyses of results from batches α1 and β1, eggs only.** The Y-axis shows the percentage of *E. multilocularis* egg spiked samples that were detected by each of four PCR methods (Taq-PCR blue diamond; EVA-PCR green square; mPCR red triangle; and CO1rtPCR purple circle), after using two different DNA extraction methods – egg sieving (dotted line, N = 24) and DNA-fishing (solid line, N = 24). The 95% confidence interval is shown for the detection level and given that all overlap; no significant differences were seen between the different methods with samples containing eggs only, with no inhibitors. The CO1rtPCR was run on α1 samples only.

All four PCR methods (Taq, EVA, mPCR and CO1rtPCR) were used to analyze DNA extracted from batch α1 whilst only three PCR methods (Taq, EVA and mPCR) were used to analyse samples in the β1 batch. The four PCR methods were able to detect a minimum 20 of the 24 positive samples containing 1-24 eggs in saline water (Figure 4). Table 1 shows how many of the samples tested positive using the different detection methods from batches α1/β1.

**Table 1:** **Results of the molecular analysis of *E. multilocularis *** **eggs-only samples using two extraction methods**

**Method**	**Egg sieving DNA extraction**		**DNA-fishing extraction**	
	**No. +ve/no. examined**	**Percentage detected [95% CI]**	**No. +ve/no. examined**	**Percentage detected [95% CI]**
**Taq-PCR**	24/24	100% [86.2-100]	22/24	91.7% [74.2-97.7]
**EVA-PCR**	21/24	87.5% [70.0-95.7]	21/24	87.5% [68.9-95.7]
**mPCR**	24/24	100% [86.2-100]	20/24	83.3% [64.1-93.3]
**CO1rtPCR**	23/24	95.8% [79.7-99.2]	Not examined	

A summary of the results of the molecular analysis of pure *E. multilocularis* egg samples using two different DNA extraction methods (egg sieving and DNA-fishing) and subsequent analysis with up to four different PCR methods (Taq-PCR, EVA-PCR, mPCR and CO1rtPCR. Three replicates were run for all methods except for EVA-PCR which was run only once due to limited material. A sample was categorised as being positive if either of the replicates produced a positive result. Egg numbers added to each sample are available in Additional file 1: Table S1.

#### α1 batch

All samples in batch α1 were positive in one or more of the three replicates^b^ tested using the four different detection methods, thus indicating successful extraction of DNA for samples containing 1-24 eggs. All 24 samples tested positive using the Taq-PCR and mPCR, 23 tested positive using the CO1rtPCR and 21 tested positive with the EVA-PCR.

#### β1 batch

None of the PCRs was able to detect all of the positive samples in the β1 batch. When interpreting that a sample was positive in at least one of three runs, the Taq-PCR successfully detected 22 of the 24 positive samples, whilst the mPCR only detected 20. The EVA-PCR was only performed as a single run but from this a total of 21 samples were positive. The number of eggs in each successfully detected sample ranged from 1-21. One sample containing 3 eggs tested false negative using all three PCR methods. It is not known why this sample failed. The CO1rtPCR was not performed on this material due to limited amounts of DNA available.

### Molecular detection on samples from batches *α2- and β2*

A total of 144 spiked fox faecal samples were investigated using one of the two DNA extraction methods, 72 using the ‘sieving’ method (*α2)* and 72 with DNA-fishing (*β2)*. The egg number in the two batches were not identical (Additional file 1: Table S1), but results of the KS test yielded a 96% probability that the number of eggs per tube between batches α2 and β2 were derived from the same distribution (Figure 2). The prepared templates of these batches were then subsequently analysed using four different PCR methods but the number of replicates varied depending on the PCR used and DNA extraction method (Figure 3).

The design of the study successfully covered the detection level of the different DNA extraction methods and the four different PCR methods. In Figure 3 the results of all the replicates are shown by DNA extraction method and PCR method and have been stratified according to the number of eggs spiked in the sample.

**Figure 5:**
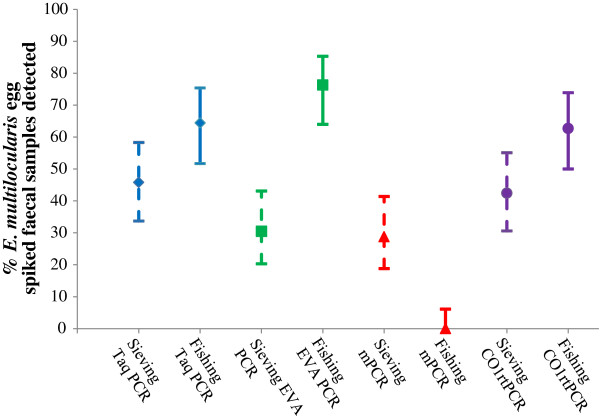
**Graph showing the percentage of *E. multilocularis *egg spiked faecal samples that were detected by each of four PCR methods (Taq-PCR blue diamond; EVA-PCR green square; mPCR red triangle; and CO1rtPCR purple circle) after using two different DNA extraction methods – egg sieving (dotted line, N = 59) and DNA-fishing (solid line, N = 59), once the negative and the high positive control samples (groups E, n = 12, and F, n = 1) were excluded from the data set.** The 95% confidence interval is given for the detection level.

**Figure 6:**
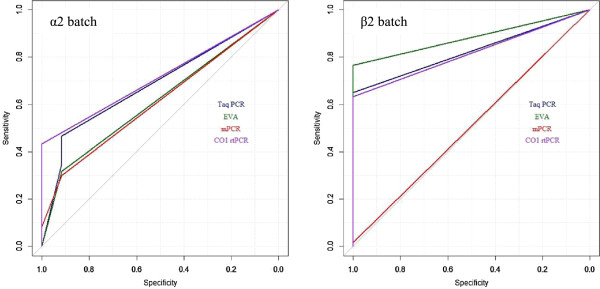
Presenting the receiver-operating characteristic curves (ROC) of the two DNA extraction methods (egg-sieving α2 and DNA-fishing β2) with the four different detection methods (mPCR, Taq-PCR, EVA-PCR and CO1rtPCR).

Looking at the results in Figure 3, it is apparent that the samples containing 15 eggs or less were less frequently detected as positive compared to the samples that were spiked with higher numbers of eggs. Figure 5 shows the percentage of samples correctly identified as containing eggs (and the 95% confidence interval) by DNA extraction method (fishing or sieving) and detection (Taq PCR, EVA PCR, mPCR, CO1rtPCR). From this figure, it is clear that the samples examined using DNA-fishing did relatively better than those investigated using egg sieving DNA extraction. All the tests were able to detect *E. multilocularis* DNA in the spiked samples, with the exception of mPCR (Figure 5). The mPCR combined with DNA-fishing was only able to detect the high positive control but none of the other samples. The overall performance recorded for the two DNA extraction protocols and the four detection methods are indicated by the plot of the ROC (Figure 6). The y-axis represents test sensitivity and x-axis represents test specificity. In general, the test sensitivity increases as test specificity decreases and the higher the area under the curve the better test accuracy. By observing the curves, the three real-time PCR methods using DNA-fishing, performed better than methods using egg sieving. Although this was not the case for mPCR, as the template from the DNA-fishing seemed not to work properly with this assay. In the β2 batch, the EVA-PCR yielded the highest sensitivity followed by Taq-PCR and CO1rtPCR, respectively, for the same level of test specificity.

In the egg sieving batch (α2) (left Figure 6), imperfect specificity (less than 1.0) was observed for EVA-PCR, mPCR, and CO1rtPCR. From our data, Taq-PCR performed the best of the PCR methods, yielding the highest sensitivity compared with other methods for the same level of test specificity (100%).

The quantitative measurement of test performance of each testing method was presented as AUC (Table 2). Overall, the AUC estimates were consistent with that observed from the ROC plots (Figure 6). Based on DNA-fishing extraction, with the exception of mPCR, the PCR methods yielded > 81% AUC, and statistically there was no significant difference in the estimates among the methods as the 95% CI for the Taq-PCR, EVA-PCR and CO1rtPCR overlapped. Based on the egg sieving extraction, the AUC estimate ranged from 61% - 71% and the 95% CI for all four PCRs overlapped.

**Table 2:** Results of the molecular analysis of the spiked fox faeces batches using four detection methods

**DNA extraction method**	**PCR method**	**Whole data set**						**Samples with ≤ 15 eggs**					
		**N**	**Se**	**Sp**	**PPV**	**NPV**	**AUC [95% CI]**	**N**	**Se**	**Sp**	**PPV**	**NPV**	**AUC [95% CI]**
Egg sieving	Taq PCR	72	0.47	0.92	0.97	0.26	0.68 [0.57-0.79]	57	0.1	0.92	0.75	0.29	0.50 [0.40-0.60]
	EVA PCR		0.32	0.92	0.98	0.37	0.61 [0.51-0.71]		0.03	0.92	0.50	0.28	0.47 [0.38-0.56]
	mPCR		0.3	0.92	0.95	0.21	0.61 [0.51-0.70]		0.07	0.92	0.67	0.28	0.49 [0.39-0.58]
	CO1rtPCR		0.43	1	1	0.26	0.71 [0.65-0.77]		0.03	1	1	0.29	0.51 [0.48-0.54]
DNA Fishing	Taq PCR	72	0.65	1	1	0.37	0.85 [0.76-0.88]	61	0.40	1	1	0.40	0.70 [0.61-0.78]
	EVA PCR		0.77	1	1	0.46	0.88 [0.82-0.93]		0.60	1	1	0.50	0.80 [0.71-0.88]
	mPCR		0.02	1	1	0.17	0.50 [0.49-0.52]		0.0	1	0.0	0.29	0.5 [0.5-0.5]
	CO1rtPCR*		0.63	1	1	0.35	0.81 [0.75-0.87]		0.3	1	1	0.37	0.65 [0.56-0.73]

Values of Sensitivity (SE), Specificity (SP), Positive predictive values (PPV) Negative predictive values (NPV) and Area under the curve (AUC) for the different DNA extractions and detection methods tested in the study. The table is divided into the whole dataset of all samples (n = 144), and separately for samples containing low numbers of *E. multilocularis* eggs (<15) (n = 84). Detection was performed in triplicate, however, due to little DNA available, *CO1rtPCR on DNA-fishing was only possible in duplicate. Egg numbers added to each sample are available in Additional file 1: Table S2.

The cut off level for the detection of false negatives was calculated for all four PCR methods and batch *α2 -*egg sieving extraction method. The Taq-PCR showed no false negative results above 76 eggs (25 eggs per gram (EPG)), and the CO1rtPCR showed no false negative results above 33 eggs (11 EPG). EVA-PCR and mPCR showed false negative results even at the maximum level of response of 140 eggs in the sample. False positive results were also detected in two of the negative control samples (Figure 4). In one sample one of the mPCR triplicate runs tested positive whilst the other two mPCR replicates were negative. The CO1rtPCR, EVA-PCR and Taq-PCR replicates also tested negative for this sample. The second false positive sample tested positive in the Taq-PCR, in all three replicates, as well as in the EVA-PCR but tested negative in the mPCR and CO1rtPCR replicates.

The cut off level for the detection of false negatives was calculated for batch *β2 -*DNA-fishing and the three real-time PCR methods. The mPCR method failed to detect eggs at low levels and only detected samples with more than 500 eggs and so was not included in the calculations. The cut off for false negative results in the replicates in the Taq-PCR data, was 26 eggs (equivalent to 8.7 EPG in the faecal sample). Similar analysis was carried out for the EVA-PCR (cut off 54 eggs, 18 EPG) and the CO1rtPCR (cut off 22 eggs, 7.3 EPG). It was possible to detect samples with eggs at less than this level but false negatives occurred especially if no duplicate or triplicate samples were investigated (Figure 4). No false positive results were detected by any of the PCRs using this method.

Results indicate a fourfold sensitivity increase, from 25 eggs per gram with the conventional mPCR and egg sieving method, compared to sensitivity of 18, 8.7 and 7.3 eggs per gram using EVA-Green, MGB-probe and ZEN-probe based real-time PCR systems combined with the DNA-fishing method. Further increased sensitivity was observed in the study when allowing for some false negatives in each run, by testing a sample in duplicate or triplicate.

## Discussion

### DNA extraction protocols –which one is best?

#### Testing of batches α1/β1 –eggs only

The batches containing eggs only were analysed prior to the batches containing faecal material since we wanted to make sure that the extraction protocols were able to handle few eggs when inhibitory material was not present. The majority of samples tested positive, which indicated that the extraction protocols worked and detection methods could be used in further testing. The testing did reveal some differences between the detection methods, but given that inhibitors were low in these samples more attention to the performance of the detection assays was given in the experiments which followed. There were more false negative samples in batch β1 compared to α1. It is possible that this might be because tubes were transported by mail prior to extraction in laboratory 2, or that the small volumes kept in the large tubes (15 ml) increased the chance of losing the eggs during the extraction procedure. False negatives were found in both batches, which indicate that there might be variations in the assays due to operator errors, or perhaps some eggs were empty containing little or no DNA for analysis. Since eggs were inactivated prior to analysis and kept in ethanol for some time it is possible that this treatment degraded DNA even more than had the eggs just been frozen.

#### Testing of batches α2/β2 –spiked faecal material

The two series (α2 and β2) which comprised of spiked fox faeces also contained other taeniid eggs, and were included to mimic a naturally occurring scenario. The mPCR results on the α2-batch indicated positive taeniid bands from all samples, proving that the extraction protocol worked. As previously reported, close to a third of foxes included in the annual surveillance programme in Norway have been shown to harbour *Taenia* sp. parasites. It is possible that these taeniid eggs and taeniid DNA could influence both the extraction protocols and the amplification steps and that the performance of the assays would have been improved if such eggs had not been present. We wanted the samples to be as realistic as possible so no *a priori* attempts were done to exclude eggs from the faecal pool used as template for the spiked samples.

The results from the screening of batches α1/β1 which contained samples containing very low egg numbers (1-5) proved successful when no faecal material was present. The introduction of faecal matter to the samples would add inhibitors to the samples. We therefore estimated that detection performance would decrease when DNA and chemical inhibitors were added to the samples. From our experiments we found that the lowest number of eggs which *could* be detected, although not consistently, from spiked fox faecal samples using either of the two DNA extraction protocols was 1. However, the methods were less robust when egg numbers were very low. Generally we found that the robustness of both the DNA extraction protocols and detection assay increased with increasing number of eggs in the samples. It was also evident that the DNA-fishing method produced extracts which gave better and more consistent results with low egg numbers. One major consideration that must be taken into account in this study that may have impacted on sensitivity is that the eggs were preserved in ethanol prior to shipping and refreezing. It is possible that the sensitivity of the assays would have been different if fresh eggs from field samples were used in the study, but this was weighed against the zoonotic risk and import consequences of using live eggs from abroad.

#### DNA extraction method - sieving versus DNA-fishing

The DNA-fishing method seem to perform better than the egg sieving method on faecal samples containing *E. multilocularis* eggs as the AUC score is generally higher for the different detection methods for these samples (Table 2). The AUC confidence intervals for the different methods for samples containing more than 15 eggs marginally overlap making them not statistical significant. However, for samples containing ≤15 eggs, the difference between the AUC intervals for the different extraction methods and real-time PCR methods do not overlap, indicating that the DNA-fishing method is superior (mPCR excluded) to the sieving method. DNA-fishing also had no false positive results. We found that the three real-time PCR detection methods were able to detect more positives using the DNA-fishing method (Figure 5). The sieving method can only detect eggs which pass through the nylon mesh leaving any adult worms, or proglottids, undetected in the faeces trapped on the first nylon mesh and discarded. Similarly if eggs were crushed during preparation, any DNA material dislodged may not be trapped on the second nylon mesh. Any eggs which may become lodged with larger material present in the faeces may also not be recovered in the first sieving procedure. The DNA-fishing extraction method has the advantage of extracting any *E. multilocularis* DNA which may be present in the sample, after thorough homogenisation of the sample, including that from any intact adult parasites, proglottids or crushed eggs.

Two samples, extracted using the sieving method, gave false positive results – one of which tested positive only in one replicate of the mPCR and the other which tested positive in all replicates of two of the real-time PCR methods. This would suggest technical error, rather than cross reactions to the PCR primers or material going from one sample to another. All equipment used during the egg sieving and DNA isolation was single-use in order to minimise the risk of cross-contamination. Despite this, due to many manual steps, such as a double sieving stage, and back flushing, this method is more vulnerable to cross-contamination than the DNA-fishing method. More automation of the various steps could decrease the chance of false positives and negatives and would thereby increase the quality of the assays. Isaksson *et al.* (in prep) have recently modified the DNA-fishing to become semi-automated using the Nordiag Bullet^c^ robot to perform some of the steps. This has further optimised the DNA-extraction protocol.

### *E. multilocularis* DNA detection methods –limitations to this study

The development of fast, sensitive and reliable assays that are economic to use are crucial for a successful surveillance programme. Although several molecular detection methods are becoming available, at the time of start of the study few real-time PCR methods for detection of *E. multilocularis* were available. This work is therefore not a review of all current diagnostic PCR methods, but a comparison of the few methods which were available to us at the onset of the project. It is possible that further optimisation of the procedures and the methods may further improve the performances of the methods used in the study.

### *E. multilocularis* DNA detection protocols –real-time PCR or multiplex PCR?

The mPCR method performed comparably to the real-time PCR methods on samples from the batches containing eggs only and with little or no inhibitors present. This was not the case once faecal material was added to the spiked samples. In batch β2 (DNA-fishing), the mPCR only detected *E. multilocularis* eggs in the sample spiked with >500 eggs and failed to detect the target DNA in any of the 59 samples in the four other groups, making it the poorest combination of methods in the study. It is not known why the template from this batch worked so poorly with the mPCR. One explanation with regard to the mPCR, is that the multiplex uses several sets of primers in the master-mix, including *Taenia* spp. These primers were able to amplify their target *Taenia* DNA, which was also present in the spiked faecal samples and was clearly visible on the gel for all samples from α2 and β2 (data not shown). These *Taenia* spp. primers may have outcompeted the specific *E. multilocularis* primers yielding more false negatives for the *E. multilocularis* specific PCR as a consequence. Data from this study indicate that real-time PCR methods generally are more suited to detect samples containing low levels of target DNA. The closed tube systems of real-time PCRs require fewer manual steps during preparation and detection thus minimizing the risk of cross-contamination and operator error.

### Real-time PCR – so which is best?

We compared three different real-time PCR’s, using samples which closely mimicked the samples which would be encountered in a surveillance programme. Taq-PCR and CO1rtPCR are both probe-based real-time PCR having reporter and quencher dyes on complementary regions of an amplified product and rely on the 5′-nuclease activity of the polymerase used in the PCR reaction to release the dyes and enable detection. EVA-PCR is a simpler system where the fluorescent dye EVA Green binds to double stranded DNA [28]. Binding of the fluorescent dye is not sequence specific. There is less risk of false positives using a probe based system than with the EVA assay due to the fact that both the primers and the probes need to be complementary to the sequence of the target for a signal to be generated. Unspecific binding of the primers to non-target DNA would therefore not generate any signal from the reaction in a probe based system. One way to decrease false positives from the EVA-PCR is to perform melting curve analysis. The probe based real-time systems limit the binding to undiscovered variants which may have enough base alterations to the target area, which would cause the primers and probe to bind more poorly increasing the risk of false negatives.

Two different probe-based real-time PCR’s were used in this study. The Taq-PCR is designed with a MGB-probe and the CO1rtPCR a ZEN™ uses a double quenched probe (Integrated DNA technologies, Coralville USA). The minor groove binder (MGB)-probes are designed to increase the melting temperature forming a more stable product and increase the sequence specificity of the probe [29]. When designing a detection assay in the mitochondrial CO1-gene, due to its A/T–richness, it may be very challenging to find a suitable area in which primers and real-time PCR probes can target, due to the thermodynamic properties of this region. A/T-rich regions cause the melting temperature to decrease, but since the double quenched ZEN™ probes can be of longer design, probe design of these difficult regions was possible.

The differences between the performances of the real-time PCR methods were marginal and difficult to differentiate, in this study. Eva-PCR had the highest AUC, but the confidence intervals of the AUCs of the different real-time PCR methods overlapped indicating that these differences were not significant. It would appear that the real-time methods in combination with the DNA-fishing extraction method are superior to the multiplex PCR and egg sieving DNA isolation. The real-time PCR methods were able to positively identify samples containing lower numbers of eggs more often than the multiplex PCR.

### The importance of replicates

We found that by increasing the replicates, more true positive samples could be detected.

In our study CO1rtPCR was only performed in duplicate^d^. From the data we have collected, we see that this PCR is already within the AUC confidence interval of the best performing methods, making it a good method. We wanted to know the influence of replicates on the AUC of the other methods by randomly choosing only two of the three available replicates. The calculations indicated that the AUC (data not shown) decreased when only two replicates were used. Hence, it is possible that the performance numbers of the rtCo1 PCR would have been improved if this method was given a full triplicate run. From the data we discovered that even fewer samples were positive from only one real-time run. This highlights the importance of running samples in duplicate or triplicates, to further increase successful detection, rather than base results from a single run. This seems to be more true especially if infection levels are suspected to be low and target DNA resides in the lower ranges of its detection limit, or if the experiments are performed manually, adding the possibility of operator errors.

## Conclusions

In endemic areas with very low infection prevalence, the frequency and intensity of *E. multilocularis* can be extremely low. The methods used in surveillance programmes should therefore be as specific and sensitive as possible and have minimal chance of generating false positive and negative results. The methods previously used in the *E. multilocularis* surveillance program in Norway, egg sieving combined with mPCR, were not as efficient as the methods assessed in this study. Based on the data presented here, mPCR seems unsuitable for detecting low levels of infection. The method selected for the Norwegian surveillance program for *E. multilocularis*, as of January 2013, was DNA-fishing and detection using real-time PCR, currently the CO1rtPCR. A second real-time PCR can also be run, using a different mitochondrial probe target if a positive sample is encountered, for verification purposes. We have implemented analysing samples in duplicate in the detection step. Two individual PCR setups minimise the risk of operator error, or a faulty PCR run, thus decreasing the chance of having false negative samples, but still keeping running costs minimal. In future studies, the molecular approaches to *E. multilocularis* detection in low infection level material described herein, should be directly compared to the SCT gold standard to assess the performance of these two approaches.

## Endnotes

^a^The protocol used for batch ‘β1’ was a shortened version of the complete protocol as these samples did not contain faeces, so many of the homogenisation and buffer steps were omitted. The complete targeted DNA extraction protocol was used only for samples in batch ‘β2’.

^b^In the Eva-PCR only one run of the samples was performed.

^c^Product now markedet as Bullet Pro® by Diasorin (Diasorin, Saluggia, Italy) (http://www.diasorin.com).

^d^For 9 samples there was only material to perform a single replicate. These samples coloured grey in Figure 3.

## Competing interests

The authors declare that they have no competing interests.

## Authors’ contributions

ØØ prepared the manuscript, designed the study, prepared spiked samples, carried out DNA extractions using sieve technique, designed one of the real-time PCR methods used in analysis, performed parts of the molecular detections and carried out analysis. MI prepared manuscript, designed study, carried out half the extractions and part of detections. SJ helped with statistical analysis and helped draft the manuscript. ÅH helped with the extraction and analysis and drafted the study. RKD helped design the study, prepared spiked samples, prepared the manuscript and data analysis. All authors read and approved the final manuscript.

## Authors’ information

All researcher work at their respective national veterinary institutes where this work was carried out to further improve the tools used in surveillance programmes carried out to assess each country’s *E. multilocularis* infection status. Methods chosen must be optimised for surveillance in low endemic countries and could therefore be different to methods commonly used in high endemic countries such as SCT or ELISA approaches. Our aim is to optimise methodology to assess if we, in Norway, have *E. multilocularis* free status with relation to the EU Directive 998/2003/EC on pet movement. The methods used in this paper have so far revealed no positive EM samples in mainland Norway since screening started in 2002 (n = 2780 samples).

## Supplementary Material

Additional file 1: Table S1 Table showing the sample identity (SID) number and the number of eggs added to the 48 samples in batches α1 and β1. **Table S2.** Table indicating the number of added eggs and the sample identity (SID) number of faecal samples from batches α2 (isolated in laboratory A) and β2 (laboratory B). Click here for file
