# Supplementary material for: Laboratory assessment of sensitive molecular tools for detection of low levels of Echinococcus multilocularis-eggs in fox (Vulpes vulpes) faeces
Source: Parasit Vectors. 2014 May 28;7:246. doi: 10.1186/1756-3305-7-246 (PMC4060867; doi:10.1186/1756-3305-7-246)
Supplement: Additional file 1: Table S1 — Table showing the sample identity (SID) number and the number of eggs added to the 48 samples in batches α1 and β1. Table S2. Table indicating the number of added eggs and the sample identity (SID) number of faecal samples from batches α2 (isolated in laboratory A) and β2 (laboratory B). [file 1756-3305-7-246-S1.docx]

**Additional file 1: Table S1**

Table showing the sample identity (SID) number and the number of eggs added to the 48 samples in batches α1 and β1.

**SID Batch # EM eggs Batch # EM eggs**

**1** α1 2 β1 2

**2** α1 3 β1 3

**3** α1 2 β1 2

**4** α1 3 B1 3

**5** α1 4 β1 4

**6** α1 3 β1 3

**7** α1 7 β1 7

**8** α1 3 β1 3

**9** α1 2 β1 2

**10** α1 2 β1 2

**11** α1 3 β1 3

**12** α1 15 β1 16

**13** α1 1 β1 1

**14** α1 8 β1 9

**15** α1 13 β1 13

**16** α1 9 β1 9

**17** α1 8 β1 8

**18** α1 24 β1 21

**19** α1 4 β1 4

**20** α1 12 β1 12

**21** α1 8 β1 8

**22** α1 13 β1 14

**23** α1 1 β1 1

**24** α1 1 β1 1

**Additional file 1: Table S2**

Table indicating the number of added eggs and the sample identity (SID) number of faecal samples from batches α2 (isolated in laboratory A) and β2 (laboratory B).

Laboratory A Laboratory B

**SID Batch #EM SID Batch #EM**

**1** α2 2 **73** β2 1

2 α2 24 **74** β2 18

**3** α2 3 **75** β2 16

**4** α2 14 **76** β2 23

**5** α2 4 **77** β2 37

**6** α2 28 **78** β2 64

**7** α2 4 **79** β2 0

**8** α2 12 **80** β2 0

**9** α2 2 **81** β2 98

**10** α2 21 **82** β2 0

**11** α2 18 **83** β2 8

**12** α2 28 **84** β2 0

**13** α2 27 **85** β2 10

**14** α2 2 **86** β2 51

**15** α2 >599 **87** β2 52

**16** α2 17 **88** β2 115

**17** α2 0 **89** β2 18

**18** α2 104 **90** β2 5

**19** α2 64 **91** β2 24

**20** α2 74 **92** β2 0

**21** α2 7 **93** β2 3

**22** α2 14 **94** β2 0

**23** α2 6 **95** β2 4

**24** α2 10 **96** β2 16

25 α2 61 **97** β2 2

**26** α2 2 **98** β2 17

**27** α2 4 **99** β2 95

**28** α2 5 **100** β2 7

**29** α2 13 **101** β2 15

**30** α2 7 **102** β2 18

**31** α2 0 **103** β2 11

**32** α2 14 **104** β2 10

**33** α2 6 **105** β2 1

**34** α2 64 **106** β2 >599

**35** α2 14 **107** β2 15

**36** α2 0 **108** β2 51

**37** α2 16 **109** β2 52

**38** α2 57 **110** β2 17

**39** α2 101 **111** β2 3

**40** α2 3 **112** β2 0

**41** α2 0 **113** β2 57

**42** α2 3 **114** β2 134

**43** α2 57 **115** β2 69

**44** α2 8 **116** β2 3

**45** α2 0 **117** β2 1

**46** α2 0 **118** β2 9

**47** α2 0 **119** β2 1

**48** α2 1 **120** β2 31

**49** α2 0 **121** β2 5

**50** α2 88 **122** β2 38

**51** α2 0 **123** β2 8

**52** α2 25 **124** β2 1

**53** α2 20 **125** β2 3

**54** α2 0 **126** β2 99

**55** α2 106 **127** β2 0

**56** α2 2 **128** β2 16

**57** α2 1 **129** β2 1

**58** α2 0 **130** β2 0

**59** α2 34 **131** β2 7

**60** α2 125 **132** β2 5

**61** α2 24 **133** β2 9

**62** α2 4 **134** β2 0

**63** α2 17 **135** β2 0

**64** α2 112 **136** β2 110

**65** α2 26 **137** β2 2

**66** α2 74 **138** β2 1

**67** α2 109 **139** β2 7

**68** α2 6 **140** β2 1

**69** α2 12 **141** β2 129

**70** α2 0 **142** β2 26

**71** α2 2 **143** β2 0

**72** α2 26 **144** β2 22
